# Immune Responses Against Classical Swine Fever Virus: Between Ignorance and Lunacy

**DOI:** 10.3389/fvets.2015.00010

**Published:** 2015-05-07

**Authors:** Artur Summerfield, Nicolas Ruggli

**Affiliations:** ^1^Institute of Virology and Immunology – IVI, Bern, Switzerland

**Keywords:** classical swine fever, macrophages, dendritic cells, virulence, interferon

## Abstract

Classical swine fever virus infection of pigs causes disease courses from life-threatening to asymptomatic, depending on the virulence of the virus strain and the immunocompetence of the host. The virus targets immune cells, which are central in orchestrating innate and adaptive immune responses such as macrophages and conventional and plasmacytoid dendritic cells. Here, we review current knowledge and concepts aiming to explain the immunopathogenesis of the disease at both the host and the cellular level. We propose that the interferon type I system and in particular the interaction of the virus with plasmacytoid dendritic cells and macrophages is crucial to understand elements governing the induction of protective rather than pathogenic immune responses. The review also concludes that despite the knowledge available many aspects of classical swine fever immunopathogenesis are still puzzling.

## Introduction

Classical swine fever (CSF) is a highly contagious disease of pigs caused by the classical swine fever virus (CSFV), which is a member of the genus pestivirus within the *Flaviviridae* family. CSFV is a spherical virus particle of 40–60 nm in diameter, consisting of a lipid envelope surrounded by a nucleocapsid packaging a positive-strand RNA genome of 12.3 kb. The RNA carries a single large open reading frame (ORF) which encodes a large polyprotein that is co- and post-translationally cleaved into the twelve proteins N^pro^, C, E^rns^, E1, E2, p7, NS2, NS3, NS4A, NS4B, NS5A, and NS5B by cellular and viral proteases. The four structural proteins C, E^rns^, E1, and E2 are components of the virion, while the others are non-structural proteins with various functions in the viral life cycle. Virus replication is restricted to the cytoplasm and does normally not result in a cytopathic effect in cell culture. Virion assembly occurs on intracellular membranes of the endoplasmic reticulum (ER), and first progeny virus is released from the cells at 5–6 h post-infection via exocytosis (1).

Classical swine fever leads to important economic losses worldwide. In Europe, the wild boar population is an important reservoir for the virus, and represents a source for reintroduction of the disease in domestic pigs.

After oronasal infection, CSFV probably passes through the epithelial cells and M-cells of the tonsilar crypts, the primary target tissue for virus replication. Thereafter, the virus is found in the tonsils and local oropharyngeal lymph nodes (2, 3). A particular affinity of the virus for the reticuloendothelial cell system has been noted with macrophages (MΦ), dendritic cells (DC), and endothelial cells (EDC) being primary targets (2–10). From these primary sites of replication, the virus spreads to other lymphoid organs. Such secondary target organs include the spleen, lymph nodes, gut-associated lymphoid tissue, bone marrow, and thymus (2, 3, 11). CSFV has also been found in the pancreas, brain, heart, gall and urinary bladders, mandibular salivary and adrenal glands, thyroid, liver, and kidney, particularly in association with EDC and MΦ (3). More recent investigations using quantitative RT-PCR confirmed these older studies, also demonstrating a wider tissue distribution with longer durations of infection (12). This is also reflected at the level of cell tropism. For example, only in late stages of the disease, viral antigen is found in peripheral lymphocytes and immature granulocytes (7, 11, 13). Furthermore, in the skin, efficient infection of keratinocytes, hair follicle epithelial cells, and mesenchymal cells in the dermis was demonstrated at later time points after infection (14).

## Highly Virulent Strains Induce a Disastrous Infection for the Immune System

### Strong peripheral and central lymphoid depletion affecting primary and secondary lymphoid tissue

The numerous field isolates and laboratory strains cover an almost continuous spectrum of virulence, from highly virulent viruses to low-virulent strains. Accordingly, the clinical outcome of CSF in pigs can vary from peracute to acute, subacute, chronic, and subclinical disease outcomes. The peracute and acute disease is characterized by pyrexia, anorexia, central nervous disorders, diarrhea, and in some cases also hemorrhages of the skin, mucosa, and various other organs. In fact, virulent CSFV can induce a typical hemorrhagic fever with immunological characteristics common to all viral hemorrhagic fevers. The disease is associated with severe lymphopenia and lymphocyte apoptosis (6, 11, 15), thrombocytopenia (3), platelet aggregation (16), bone marrow depletion affecting myelopoiesis and magakaryocytopoiesis (11, 17), and thymus atrophy as well as thymocyte apoptosis (5, 13). Lymphoid depletion is generalized, not only affecting peripheral blood and lymph nodes but also the mucosal tissue (18). At later stages, disseminated intravascular coagulation (DIC), petechial bleedings, and hemoconcentration can be found (3), which can result in a circulation failure, hypotension, and death. A recent study, however, suggests that the hemorrhagic lesions observed in the late stages of the disease are not attributable to DIC. Inhibition of diffuse fibrin and thrombi formation did not influence the extent of hemorrhagic lesions. From this, it was concluded that DIC was not the cause for the thrombocytopenia and hemorrhages observed in acute–lethal CSF (19).

### Massive induction of interferon-α

**Figure 1:**
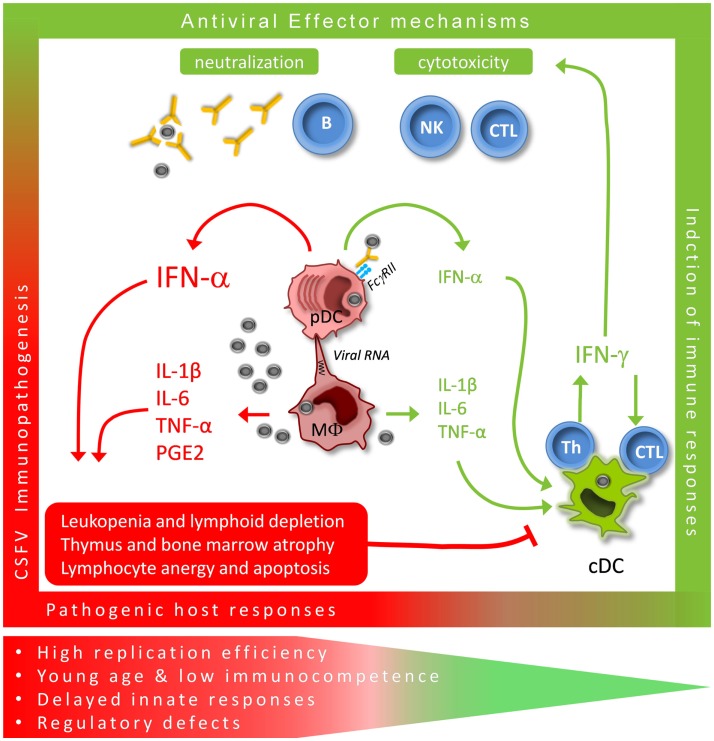
**Critical immunological pathways for protective (green) versus pathogenic (red) immune responses during acute CSFV**. CSFV targets both monocytic cells with their MΦ descendants and conventional and plasmacytoid DC (cDC and pDC). MΦ are mainly responsible for the typical pro-inflammatory responses, although conventional DC may contribute to this response. We propose that the large quantities of IFN-α produced by pDC play a central role in the innate immune response to CSFV. Prolonged systemic responses are associated with pathogenic host responses while time-limited production appears to promote protective adaptive Th1 effector responses.

Very high levels of serum interferon-(IFN)-α are a hallmark of the acute disease phase induced by virulent CSFV. It appears that the levels of IFN-α found in the serum correlate with disease severity and the virulence of the isolate used for infection (20, 21). Nevertheless, the association between virulence and high IFN-α levels was less clear in 6-month-old pigs (22). Our experience in younger animals clearly indicated a correlation between serum IFN-α levels and the degree of lymphopenia induced by CSFV. In fact, the onset of severe lymphopenia was concomitant with the IFN-α responses, and all animals with serum IFN-α had depleted peripheral B and T lymphocytes (21). These observations indicate that high levels of IFN-α cannot control the virus but may rather mediate aberrant responses leading to immunopathology (Figure 1). Microarray analyses of PBMC isolated from infected pigs confirmed not only the dominance of IFN-stimulated genes but also of cell death receptor and apoptosis pathways such as TRAIL, FAS, and TNF (23), relating to previous studies performed with peripheral blood cells using flow cytometry (15). To our knowledge, compared with other virus infections of pigs, CSFV can induce not only the most long-lasting but also the most intense systemic IFN-α responses (24).

### Infection and activation of MΦ

*In vivo*, MΦ infection and morphological signs of activation were found in the spleen (4, 25), the kidney (26) the lung (27), the liver (28), and the intestine (18). In addition, infection of pigs was associated with MΦ producing pro-inflammatory cytokines, such as IL-1α, IL-1β, IL-6, and TNF-α (5, 8, 25, 28). There is also evidence for macrophage activation leading to the production of vasoactive mediators including prostaglandin E2 (8) and platelet activation (16). Finally, during acute and severe CSF, an activation of the complement system has been observed (5, 25). Therefore, MΦ infection and activation have been proposed to play an important role in CSF pathogenesis, in particular, through release of pro-inflammatory and vasoactive mediators (Figure 1).

### Infection and activation of dendritic cells

It was also shown that CD11R1^+^CD172a^+^ cells, probably representing a subset of conventional DC (29), are activated *in vivo* in the blood, the tonsil, and the spleen at 24–48 h post-infection to produce TNF-α and IL-10 (10). The same study also demonstrated IFN-α and IL-12 producing CD4^+^CD172a^+^ plasmacytoid DC (pDC) in the same immunological compartments but possibly with an ever faster kinetic of response. Immunofluorescence analysis indicated that these two populations of DC do represent early target cells of CSFV (10). Only recently it became clear that the monocytic cells and DC represent two distinct lineage of cells with respect to their ontogenic development (30), and it is now also possible to clearly differentiate bona fide DC from monocytic cells in the pig (29). Although these cells do have overlapping functions, they also have a clear functional specialization. During CSF, MΦ are probably mainly responsible for the typical pro-inflammatory responses, but conventional DC appear to contribute to this response although they may also be involved in counteracting it by secretion of anti-inflammatory IL-10 (10). Finally, pDC typically secrete large quantities of IFN-α, but possibly also Th1-promoting IL-12. The impact of the virus on the antigen-presenting functions of DC is not clear but it appears that the cells are not depleted in the lymphoid tissues at least in the first 2–3 days post-infection (10).

### Effects on lymphocytes

Despite the severe lymphoid depletion, acute CSF is also associated with a pronounced anergy of T lymphocytes in the acute phase of the disease (13, 15). At later stages of severe CSF, T cell activation events (31, 32) with the detection of serum IL-2 and IFN-γ (33) have been found. Similarly, indication of B-cell activation has been described in terms of an increase in cells expressing the lambda light chain and IgM (34).

## Low-Virulent Strains: From Controlled to Chronic Infections

In contrast to severe forms of the disease described above, infection with low-virulent strains of CSFV induces no obvious clinical symptoms or only weak and transient disease. In the serum of such animals, no or lower levels of IFN-α and pro-inflammatory cytokines can be detected (21, 22, 35). However, these animals often also develop transient lymphopenia (36, 37). If controlled, such infections result in life-long immunity against CSFV. Nevertheless, depending on the age and immune status, infection with low or moderately virulent CSFV may lead to forms of chronic disease, which can last up to 3 months before the animals die (38–40). Due to the inability of the immune system to clear the infection, these animals shed large quantities of virus and play an important role in epidemiology of the disease (41, 42). Initially, the immunopathogenic events in such animals can be similar to those described above albeit milder. At later stages, signs of lymphocyte activation and proliferation are found, which are not well defined (43, 44).

## Virus–Host Interactions at the Cellular Level

### CSFV proteins targeting innate immune responses

#### N^pro^

**Figure 2:**
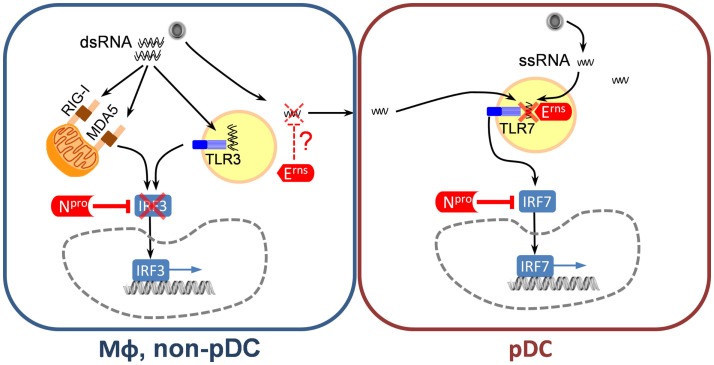
**Classical swine fever virus-encoded inhibitors of the IFN type I system**. In MΦ and non-pDC target cells, N^pro^ represents the main IFN antagonist, which almost completely inhibits IRF3-mediated IFN type I induction induced by sensing viral dsRNA via RIG-I, MDA-5, and/or TLR3. In pDC, N^pro^ is also active by inhibiting IRF7-mediated IFN-α induction although this inhibition is not complete. In addition, E^rns^ represents a potent inhibitor of pDC responses through its ability to degrade viral ssRNA and thereby prevent TLR7 activation. Viral ssRNA can originate from virus replicating in pDC or in neighboring cells. The mechanism of viral RNA transfer as well as the subcellular location of RNA degradation is not clear.

The N^pro^ is a cysteine autoprotease that cleaves itself from the viral polyprotein co-translationally and targets IRF3, an essential transcription factor for *IFNB1*. The C-terminal half of N^pro^ carries a zinc-binding domain that is required for interaction with IRF3 (45, 46). Through this interaction, N^pro^ induces efficient proteosomal degradation and depletion of IRF-3, which is the basis of the very potent antagonism of IFN type I induction by CSFV (47–51) (Figure 2). However, pDC are unique by constitutively expressing IRF7, and in contrast to other cells do not require IRF3 for induction of IFN-α/β (52), explaining why this cell type is exclusively able to respond to CSFV by IFN-α/β production. Nonetheless, N^pro^ was found to be also partially active in pDC, presumably through its ability to interact also with IRF7 and prevent IRF7-mediated IFN type I induction (53) (Figure 2). Furthermore, also in GM-CSF-driven bone marrow hematopoietic cell-derived DC, which have been induced to express IRF7 by IFN type I pre-treatment, N^pro^ was still inhibitory (54). In addition to its ability to suppress IFN type I responses, N^pro^ also mediates anti-apoptotic effects induced by synthetic double stranded (ds) RNA but not by FasL or staurosporine (48, 55, 56), preventing activation of caspases 8, 9, and 3 and inhibiting the loss of mitochondrial membrane potential and cytochrome c release (48, 55, 56). Interestingly, N^pro^ interacts with the anti-apoptotic HS-1-associated protein X-1 (HAX-1), inducing the redistribution of HAX-1 to the ER compartment. This HAX-1 redistribution to the ER during CSFV infection may increase cellular resistance to apoptosis, similar to other HAX-1 interacting proteins (56). N^pro^ also interacts with IκBα known to prevent NFκB p65 nuclear translocation but this interaction apparently has no impact on NFκB translocation (57).

Studies in pigs indicate that the N^pro^-mediated interference with IFN type I induction contributes to pathogenicity. Single amino acid mutations specifically eliminating the ability of N^pro^ to interact with IRF3 partially attenuated moderately virulent but not highly virulent CSFV (20). On the other hand, reintroduction of functional N^pro^ into moderately virulent GPE^−^-derived virus with unfunctional N^pro^ enhanced virulence by preventing IFN type I induction at local replication sites (58).

#### E^rns^

This essential structural glycoprotein of pestiviruses has a remarkable RNase activity, with structural similarities to plant T2 RNases. The optimal catalytic activity is at acidic pH (59), with preferential cleavage of single-stranded (ss) RNA (60, 61). The protein also has an unusual membrane anchor composed of an amphipathic helix without a typical membrane anchor (62, 63), but a retention signal ensuring its association with the intracellular membrane compartments (64). Based on the observation that a minor fraction of the protein was found to be secreted from infected cells or cells expressing E^rns^ (62, 64, 65), a role for secreted E^rns^ acting in the extracellular compartment where it could degrade RNA has been postulated (66–68). In addition, E^rns^ can be rapidly endocytosed to also degrade endosomal RNA in adjacent cells (69). However, these studies were performed with recombinant E^rns^. In the viral context, the antagonistic activity of E^rns^ on IFN-α induction was only demonstrated in pDC (70). On one side, CSFV expressing E^rns^ lacking RNase activity in contrast to wild-type virus was able to induce very strong IFN-α responses in pDC. On the other hand, cells infected with virus replicon particles lacking E^rns^ or CSFV expressing an E^rns^ without RNase activity were much more efficient at stimulating pDC than cells infected with the parent virus. This very potent stimulation of pDC by infected cells was demonstrated not to be mediated by virions but by a transfer of viral RNA to the TLR7 compartment of pDC. Based on this data it can be concluded that E^rns^ degrades viral ssRNA preventing its interaction with TLR7 in pDC. Considering that the RNase activity of E^rns^ is particularly high at acidic pH, an attractive model is that degradation would happen in the endosomal compartment (70) (Figure 2). We consider these findings as relevant since the stimulation of pDC by infected cells results in much higher levels of IFN-α as compared to the direct pDC stimulation by virions (70). The role of E^rns^ in other cell types expressing TLR7 and TLR8 or even TLR3 such as monocyte/MΦ, B cells, and other DC subsets still needs to be investigated. The first step would be to characterize the TLR expression in pigs. *In vivo* removal of the RNase activity results in virus attenuation (71) and abrogation of the capacity of pestiviruses to establish immunotolerance and persistent infection after infection of fetuses (72). The relationship between this inhibitory activity of Erns on the innate immune responses mediated via TLR7 and the establishment of tolerance will be an important area of future investigations.

### Quiescent *in vitro* infections

With a few exceptions, CSFV is absolutely non-cytopathogenic. In all target cells analyzed so far, except pDC, the virus does not induce IFN type I responses, and no or low cytokine responses are found. This is independent of the virulence of the strains investigated. MΦ activation following *in vitro* infection with CSFV is surprisingly weak compared to stimulation with TLR ligands such as lipopolysaccharide, and many reports confirm that CSFV only induces a minor activation of monocytic cells including monocyte-derived MΦ, monocyte-derived DC, as well as their bone marrow-derived counterparts (8, 54, 73, 74). Zaffuto et al. (75) reported microarray data showing only 11 out of 7712 genes (0.14%) induced by the virus, including arginase-1, phosphoinositide 3-kinase, chemokine receptor 4, and interleukin-1β. Obviously, these characteristics are dependent on the potent ability of N^pro^ to counteract virus sensing (76). Previous work also demonstrated that CSFV neither induces nor interferes with NFκB signaling (57). These reports with cell lines, monocyte-derived cells, and *ex vivo* isolated macrophages are remarkable, considering the replicative ability of the virus in these cells. This demonstrates that the viral innate immune system antagonist N^pro^ is most efficient in hiding the infection. For pestiviruses, it is well known that the balance of viral dsRNA accumulating during replication is regulated by a tightly controlled expression of NS3 (77). Cytopathogenic mutants typically have higher levels of dsRNA. Accordingly, such mutants do induce IFN-β and activate monocytic cells even with functional N^pro^, indicating that evolution has driven a well-balanced relationship between N^pro^ and viral dsRNA (76). In fact, using non-functional N^pro^ mutants of CSFV, we have demonstrated that in PK-15 cells viral RNA is sensed by TLR3, RIG-I, and MDA-5 (78).

### Plasmacytoid DC responses *in vitro*

Classical swine fever virus activates pDC to produce IFN-α. This activation requires live virus and pDC infection (79). Nevertheless, compared to other viruses such as influenza virus the levels of IFN-α are relatively low (24). In fact, this can be explained by the action of N^pro^ targeting IRF7 (53) and of E^rns^ degrading viral RNA to prevent the triggering of TLR7 by viral RNA (70). A very efficient TLR7-dependent induction of IFN-α in pDC by CSFV-infected cells in the absence of virions has been demonstrated. This pathway is mediated by a transfer of RNA from an infected donor cell to pDC in a cell contact-dependent manner requiring intact lipid rafts and cytoskeleton of the donor cell. E^rns^ blocks both direct stimulation of pDC by virions and stimulation by infected cells (70). Although on a per cell basis CSFV is a weak activator of pDC, its strong tropism for lymphoid tissue and pDC is likely to result in the overall high and prolonged responses found *in vivo* (24).

## Proposed Mechanisms Leading to Control

### Dysregulated responses

Published data indicate that at the initial sites of virus replication – involving principally MΦ and epithelial cells – CSFV N^pro^ inhibits virus-induced IFN-α/β allowing the virus to replicate and generate the virus load leading to viremia and spread within the organism. The speed and level by which CSFV replicates and spreads appears to be critical for the outcome of disease. The virus then infects more MΦ and pDC, resulting in massive IFN-α and pro-inflammatory cytokine release as described above (Figure 1). Based on the known effects of IFN-α/β on MΦ activation it is tempting to postulate that pDC activation may enhance these effects. Nevertheless, to our surprise even IFN-primed MΦ did not respond to CSFV by production of IFN-α, IL-1β, or IL-6 production (54). It is thus still puzzling to observe the discrepancy between *in vitro* and *in vivo* with regards to MΦ activation.

In vaccinated and immune animals, there are no or less immunopathological events such as development of leukopenia and systemic inflammatory responses after challenge infection (80–82). However, vaccinated animals still respond to CSFV infection with a serum IFN-α response, even in absence of viremia, but, in contrast to naïve animals, this response is lower and only of short duration (79–81, 83). A possible explanation for this observation is the fact that pDC from vaccinated pigs carry cytophilic antibodies, which mediate efficient capture of CSFV, resulting in early strong pDC stimulation (79). This observation underlines that in contrast to strong long-lasting systemic IFN-α responses, a short-lived IFN-α response is probably beneficial for the immunity against CSFV. *In vivo* administration of high levels of IFN type I is known to have comparable negative effects on the hematopoietic system (84–87). Moreover, when IFNAR knockout mice were employed in a lymphocytic choriomeningitis virus model, no induction of hematopoietic cell depletion and leukopenia was observed (88). In fact, the known antiproliferative and proapoptotic effects of IFNs (89) could be directly responsible for hematologic cytopenia (Figure 1).

Several attempts to shed light into host responses related to control of CSFV by the immune system have used transcriptomic profiling (23, 90, 91). In response to CSFV infection, increased expression of IFN-stimulated genes as well as other immune response genes, genes related to cell cycle, apoptosis, metabolism, and others were observed. The profiles described reflected what was expected in terms of IFN and cytokine responses measured by ELISA, apoptosis of lymphocytes, and general changes in immune cell composition described for CSF. Using a moderately virulent strain of CSFV, Hulst and co-workers compared groups of pigs able to control the infection with those developing chronic disease and excreting high quantities of virus over a period of 35 days (92). Interestingly, the animals that recovered later had a generally more robust early response in terms of genes associated with IFN type I responses and macrophage activation, whereas those developing chronic disease were found to express inhibitors of the NFκB pathway. This study also indicated a dysregulation of the complement cascade and the vitamin D3 metabolism in animals not controlling the infection. On the other hand, this work also showed that immunoregulatory molecules such as indoleamine 2,3-dioxygenase 1 (IDO1) were expressed early in controller pigs but late in non-controllers (92). Certainly, such analyses highlight the complexity of protective immune responses, which are composed of both stimulatory and regulatory elements required to prevent tissue damage at the right moment. It appears that this is a central theme in understanding the complex pathogenesis of CSF (Figure 1).

### Protective immune mechanisms

It is well established that conventional live attenuated CSFV vaccines have an extraordinary protective capacity inducing protection as early as 3–5 days post vaccination (93, 94). Remarkably, transmission can even be prevented when animals are vaccinated on the day of challenge (95). Obviously, this protection is found in complete absence of neutralizing antibodies indicating alternative mechanisms of protection early post vaccination.

For this reason, several groups investigated the potential role of IFN-γ-secreting T cells (32, 35, 94, 96–98). Typically after challenge infection of pigs with virulent CSFV, only the previously vaccinated animals had circulating T cells secreting IFN-γ. In contrast, vaccination alone using C-strain based vaccine was not efficient at inducing detectable levels of activated peripheral T cells. Only in one study using three shots of a DNA vaccine, CSFV-specific IFN-γ spots were also found before infection (98). Also, in unvaccinated animals, which are challenged with a virulent strain of CSFV, no T cell activity can be detected in the peripheral blood. This is certainly caused by the severe defects in their T cell compartment which is even unable to respond to polyclonal stimulation (13, 15). Most of the IFN-γ-producing lymphocytes found in the peripheral blood belong to the CD4^−^CD8β^+^ T cell subset and co-express perforin indicating effector functions (82, 97) and are probably effector CTL’s since they express CD107a on their surface. This is in line with previous work demonstrating cytotoxic T cell activity against CSFV-infected target cells (96). From the latter study, it appears that the ability to detect CSFV-specific cytotoxic T cell activity requires a certain level of virus replication; since in this study, CTL activity was found only in the peripheral blood of animals kept unvaccinated but challenged with a moderately virulent virus. A recent report showed induction of MHC class II on NK and γδ T cells by IFN-α derived from CSFV-infected pDC *in vitro*. However, *in vivo* this was only found in tonsils and retropharygeal lymph nodes of pigs infected with virulent virus, but not following vaccination with attenuated vaccines. Furthermore, neither an increase in perforin nor IFN-γ was found both *in vitro* and *in vivo*. From this, the authors concluded that these cell types are probably not contributing to early protection induced by attenuated vaccines (99).

These studies showing an association of T cell responses with protection alone do not permit a conclusion that IFN-γ secreting T cells are a correlate of protection or even have protective value and the general contribution of T cells to protection remains unclear. A main problem is the immunopathological effects of CSFV on the T lymphocyte compartment, which if present do not permit the detection of any T cell activation. Furthermore, NS3 protein known to contain T cell epitopes (100) was not able to confer partial protection in vaccination-challenge studies (101, 102) indicating that T cell immunity alone is unlikely to control CSFV. On the other hand, neutralizing antibodies against E2 are well known to be associated with protection (83, 103–106). But E2 also contains CTL epitopes (107) whose contribution to protection is not yet clear.

## Conclusion and Future Research

Despite the knowledge available on the pathogenesis of CSF, many essential aspects remain enigmatic and can only be clarified with well-defined gain- as well as loss-of-function *in vivo* experimental models. In our view, the most important questions are to identify the precise contribution of various cell types including pDC, conventional DC, and MΦ to disease pathogenesis and immunity both during the acute forms of CSF and during chronic disease. Similarly, correlates of early vaccine-induced protection remain unproven. The functions of various subsets of T cells in protection need to be defined to understand both chronic disease and vaccine-mediated early protection. Furthermore, the exact role of evolvement of viral inhibitors of the IFN type I system, targeting both non-pDC and pDC remains puzzling considering that *in vivo* IFN-α/β responses are induced. Finally, the cellular systems and the viral inhibitors described in this review need to be understood in the light of the ability of pestiviruses to induce immunotolerance, if infection occurs during certain stages of the development of the immune system.

## Conflict of Interest Statement

The authors declare that the research was conducted in the absence of any commercial or financial relationships that could be construed as a potential conflict of interest.
